# The Evolutionary Consequences of Blood-Stage Vaccination on the Rodent Malaria *Plasmodium chabaudi*

**DOI:** 10.1371/journal.pbio.1001368

**Published:** 2012-07-31

**Authors:** Victoria C. Barclay, Derek Sim, Brian H. K. Chan, Lucas A. Nell, Maia A. Rabaa, Andrew S. Bell, Robin F. Anders, Andrew F. Read

**Affiliations:** 1 Center for Infectious Disease Dynamics, Department of Biology, The Pennsylvania State University, University Park, Pennsylvania, United States of America; 2 Department of Biochemistry, La Trobe University, Melbourne, Australia; 3 Department of Entomology, The Pennsylvania State University, University Park, Pennsylvania, United States of America; 4 Fogarty International Center, National Institutes of Health, Bethesda, Maryland, United States of America; Stanford University, United States of America

## Abstract

Malaria vaccine developers are concerned that antigenic escape will erode vaccine efficacy. Evolutionary theorists have raised the possibility that some types of vaccine could also create conditions favoring the evolution of more virulent pathogens. Such evolution would put unvaccinated people at greater risk of severe disease. Here we test the impact of vaccination with a single highly purified antigen on the malaria parasite *Plasmodium chabaudi* evolving in laboratory mice. The antigen we used, AMA-1, is a component of several candidate malaria vaccines currently in various stages of trials in humans. We first found that a more virulent clone was less readily controlled by AMA-1-induced immunity than its less virulent progenitor. Replicated parasites were then serially passaged through control or AMA-1 vaccinated mice and evaluated after 10 and 21 rounds of selection. We found no evidence of evolution at the *ama-1* locus. Instead, virulence evolved; AMA-1-selected parasites induced greater anemia in naïve mice than both control and ancestral parasites. Our data suggest that recombinant blood stage malaria vaccines can drive the evolution of more virulent malaria parasites.

## Abstract 2

A candidate malaria vaccine promoted the evolution of more virulent malaria parasites in mice.

## Author Summary

Vaccination can drive the evolution of pathogens. Most obviously, molecules targeted by vaccine-induced immunity can change. Such evolution makes vaccines less effective. A different possibility is that more virulent pathogens are favored in vaccinated hosts. In that case, vaccination would create pathogens that cause more harm to unvaccinated individuals. To test this idea, we studied a rodent malaria parasite in laboratory mice immunized with a component of malaria vaccines currently in human trials. We found that a more virulent parasite clone was less well controlled by vaccine-induced immunity than was its less virulent ancestor. We then passaged parasites through sham- or vaccinated mice to study how the parasites might evolve after multiple rounds of infection of mouse hosts. The parasite molecule targeted by the vaccine did not change during this process. Instead, the parasites became more virulent if they evolved in vaccinated hosts. Our data suggest that some vaccines can drive the evolution of more virulent parasites.

## Introduction

Evolution is a significant challenge to malaria control. Malaria parasites have repeatedly evolved resistance to frontline drugs [1],[2], and mosquitoes have evolved resistance to all classes of approved insecticides [3],[4]. Here we report experimental studies investigating how malaria parasites might evolve in response to the “natural” selection imposed by a blood stage malaria vaccine. There is currently no licensed malaria vaccine, but a number of candidates are in human trials [5]–[9], and a vaccine targeting the pre-erythrocytic stages of *Plasmodium falciparum* has provided partial protection to young children in a large phase 3 trial in Africa [10].

There are two ways parasites could evolve in vaccinated populations. Vaccine developers have traditionally been concerned with epitope evolution (antigenic escape) [5],[8],[9],[11],[12]. This is where pre-existing or *de novo* variants of target antigens emerge and spread because they enable parasites to evade vaccine-induced immunity. Epitope evolution in response to vaccination occurs in a range of infectious agents, including hepatitis B virus [13],[14], *Bordetella pertussis* [15]–[18], and *Streptococcus pneumoniae* [19],[20]. Epitope evolution has been of particular concern for those developing blood stage malaria vaccines because target antigens are often highly polymorphic, presumably because of natural immune selection. Considerable ingenuity is currently going towards inducing variant-independent immunity against these targets [7],[21]–[27].

Epitope evolution is not the only type of evolution that can occur in response to vaccination. Immunization can also promote the emergence of variants at loci other than those targeted by vaccine-induced immunity [28]. Of particular interest are virulence determinants because, in theory, immunization can under some circumstances promote the emergence and spread of strains causing more severe disease (morbidity and mortality) [28]–[37]. The idea that vaccines could prompt the evolution of more virulent pathogens is controversial, but it has been described as one of the key unexpected insights to arise from the nascent field of evolutionary medicine [38]. Several veterinary vaccines have failed in the face of more virulent strains, apparently in the absence of epitope evolution [39]–[43].

Vaccination could favor virulent malaria parasites in two ways. First, if the primary force preventing the evolution of more virulent strains is that they kill their hosts and therefore truncate their infectious periods, keeping hosts alive with vaccination will allow more virulent strains to circulate [28]–[37],[44]. Second, immunity might be less effective against virulent strains [36]. For instance, a given antibody titer or a proliferating immune response might better control slower replicating strains than more aggressive strains [45]. Virulence factors that reduce the efficacy of primed immune responses might also have a selective advantage in vaccinated hosts [46].

Epitope evolution and virulence evolution are not necessarily mutually exclusive (some antigens can be virulence determinants), but they will have different consequences for public and animal health. Epitope evolution will erode vaccine efficacy but need not lead to more severe disease in unvaccinated individuals. Virulence evolution on the other hand would both erode vaccine efficacy and cause more severe disease outcomes in unvaccinated individuals [28],[35],[36]. Note that virulence evolution will not occur for vaccines that induce sterilizing immunity: evolution can proceed only where vaccines are leaky so that wild-type pathogens can transmit from vaccinated hosts. Because natural immunity against malaria is neither life-long nor sterilizing [47],[48], it seems likely that malaria vaccines will be leaky.

To investigate the consequences of blood stage malaria vaccination for epitope and virulence evolution, we performed serial passage experiments with the rodent malaria *Plasmodium chabaudi* in laboratory mice immunized with a candidate blood stage vaccine. In this system, virulence, which we measure as weight loss and particularly anemia, is positively related to transmission and competitive ability [35],[36]. Anaemia is due to direct red cell destruction by parasites and bystander killing by host responses [35],[36],[49]. As with many pathogens [50],[51], serial passage of *P. chabaudi* creates more virulent parasites [52]. Serial passage through mice immunized with live parasites augments this effect [30], consistent with the idea that parasites evolving in vaccinated populations could become more virulent. However, most probably, actual blood stage vaccines will consist of recombinant antigens [53]–[69]. Here we specifically test the evolutionary impact of vaccination with Apical Membrane Antigen-1 (AMA-1), a component of at least 10 vaccines in human trials [6],[66]–[68]. Antibodies elicited by this antigen are believed to confer protection by inhibiting the invasion of merozoites into red blood cells (RBCs) [55],[65],[69]. In nature, the *ama-1* gene is highly polymorphic, and this antigenic diversity is thought likely to compromise vaccine efficacy in the long term [7],[70]–[72]. By immunizing with a highly defined single recombinant blood stage antigen, we could specifically determine whether antibodies raised against AMA-1 select for parasites with altered *ama-1* sequence (epitope evolution) and/or for parasites that cause more severe disease (virulence evolution). We found no evidence of epitope evolution in response to vaccination, but virulence increased.

## Results

Our experimental evolution studies consisted of two serial passage experiments, denoted A and B, and four separate “evaluation” experiments to determine the virulence of the passaged lines, denoted experiments 1 to 4 (Table S1).

### Serial Passage Generates Virulent Parasites That Are Less Well Controlled by AMA-1 Vaccination

Before beginning experimental evolution in vaccinated animals, we wanted to test whether AMA-1 vaccine-induced immunity would be less effective against virulent parasites. In order to generate virulent parasites, we serially passaged a single clonal lineage of *P. c. adami* (clone DK) through 30 successive naïve mice (“serial passage A”). We then tested the performance and virulence of these virulent parasites and their less virulent ancestral precursors in sham- and AMA-1-vaccinated mice (“evaluation experiment 1”).

**Figure 1:**
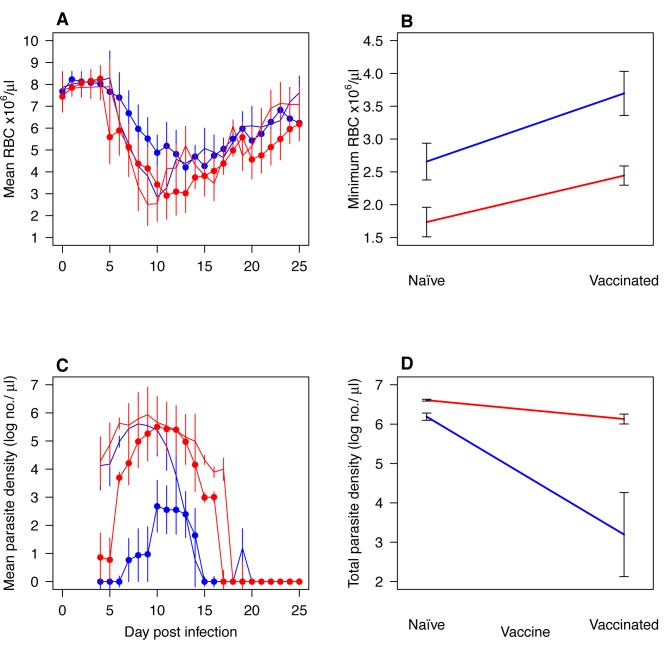
Virulence and densities of *P. c. adami* parasites that had undergone 30 passages in naïve mice (derived) with their progenitors (ancestral) (“evaluation experiment 1”). Curves (panels A and C) represent the kinetics in up to four mice (mean ± 1 s.e.m.) that were sham-vaccinated (no symbols) or AMA-1 vaccinated (filled circles) and infected with derived (red) or ancestral parasites (blue). Interaction plots (panels B and D) show minimum parasite densities and red cell densities in sham- or AMA-1-vaccinated mice infected with ancestral parasites (blue lines) or derived parasites (red lines). Derived parasites induced more anaemia and achieved higher parasite densities than ancestral parasites during infection of naïve mice (A–D; anemia *F*_1,6_ = 6.5, *p* = 0.04, parasites *F*_1,6_ = 22.3, *p* = 0.003) and AMA-1 vaccination was disproportionately less effective at containing the derived parasites (C–D; total parasite density×vaccination: *F*_1,12_ = 5.4, *p* = 0.03).

As expected, serial passage produced parasites that were more virulent in naïve mice than were the ancestral parasites (Figure 1A–B; anemia *F*_1,6_ = 6.5, *p* = 0.04). Vaccination with recombinant AMA-1 reduced anemia (Figure 1A–B). It also suppressed parasite densities (Figure 1C–D). Importantly, vaccine-induced immunity was disproportionately effective at containing the avirulent (ancestral) parasites, even though they shared complete sequence identity at *ama-1* with the more virulent (derived) parasites (Figure 1C–D; total parasite density×vaccination: *F*_1,12_ = 5.4, *p* = 0.03). This suggests that AMA-1 vaccination has the potential to selectively favor more virulent *P. chabaudi* parasites. Serial passage did not affect the nucleotide sequence of *ama-1* (Figure S1).

### Serial Passage through Vaccinated Mice Caused Enhanced Virulence, Not Target Site Evolution

To test the evolutionary impact of vaccination with AMA-1, we contemporaneously passaged *P. c. adami* DK parasites every week for 20 wk through either sham-vaccinated mice or through mice vaccinated with recombinant AMA-1 (“serial passage B”). We refer to the parasite lines evolved under these contrasting conditions as C-lines and V-lines, respectively. We set out to evolve five independent replicate lines of each type, but particularly in vaccinated groups, lineage loss occurred when parasites failed to reach high enough densities to allow onward syringe passage. Failure to achieve transmissible densities in vaccinated hosts is likely to be an important evolutionary force. When lines were lost, sub-lines were derived from surviving lines. The full evolutionary history of the lines is shown in Figure S2.

Throughout the 20 passages, parasite densities on the day of passage were lower in AMA-1 vaccinated mice (Figure S3). However, the densities of those V-lines increased steadily over the successive passages, presumably because of parasite adaptation to vaccine-induced immunity.

To test whether parasite virulence had evolved during the passages, we evaluated the virulence of the parasite lines in naïve mice at two time points during the evolution of the lines: once after 10 rounds of serial passage (“evaluation experiment 2”) and again after 21 rounds (“evaluation experiment 3”). In that latter experiment, we also assayed the virulence of the ancestral parasites (passage 0). We used naïve mice in these experiments because the hypothesis under test is that evolution through AMA-1 vaccinated mice will produce parasites that do more harm to unvaccinated hosts.

**Figure 2:**
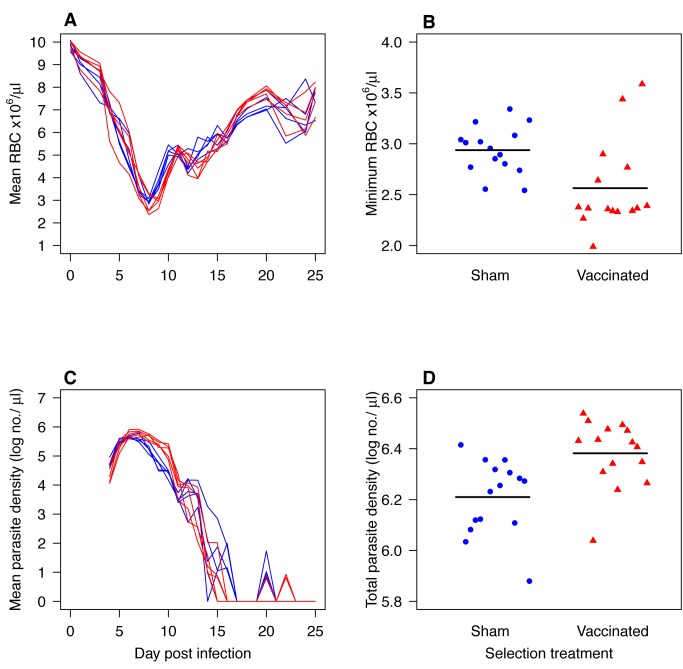
Virulence and densities in naïve mice of parasites that had previously been serially passaged 10 times through mice that were sham-vaccinated or AMA-1 vaccinated (“evaluation experiment 2”). Curves (A and C) show the kinetics of five C-lines (blue) and five V-lines (red) each assayed in up to three mice. Points on the scatterplots (B and D) are individual mice infected with C-lines (filled blue circles) or V-lines (filled red triangles). Horizontal black lines indicate mean values. V-lines induced more anemia (A–B; *F*_1,28_ = 8.4, *p* = 0.007) and reached higher total parasite densities than their comparator C-lines (C–D; *F*_1,28_ = 11.5, *p* = 0.002).

**Figure 3 pbio-1001368-g003:**
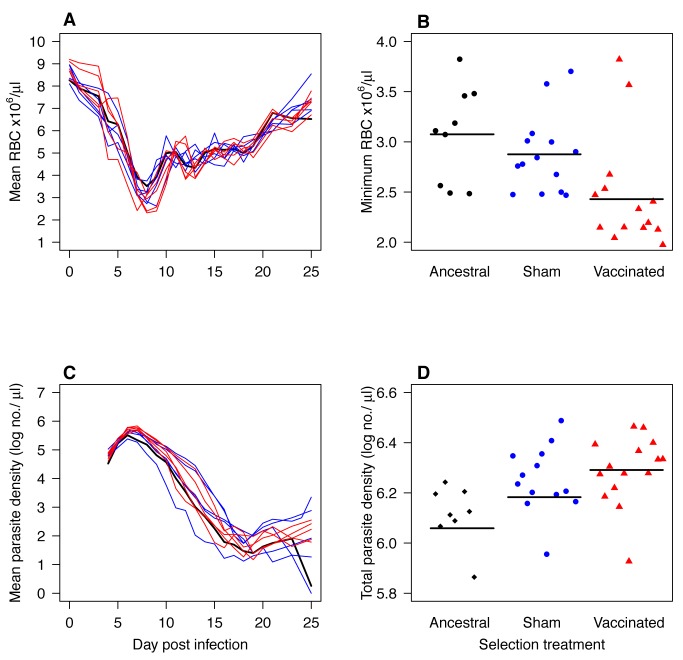
Virulence and densities in naïve mice of parasites that had previously been serially passaged 21 times through mice that were sham-vaccinated or AMA-1 vaccinated, together with the progenitor parasites (ancestral) (“evaluation experiment 3”). Curves (A and C) show the kinetics of five C-lines (blue) and five V-lines (red) each assayed in up to three mice. Black curve is the mean of nine mice infected with the ancestral lineage. Points on the scatterplots (B and D) are individual mice infected with ancestral parasites (filled black diamonds), C-lines (filled blue circles), or V-lines (filled red triangles). Horizontal black lines indicate mean values. V-line parasites caused more anemia than the C-lines and ancestral parasites (A–B; *F*_1,27_ = 6.2, *p* = 0.02 and *F*_1,22_ = 8.2, *p* = 0.008, respectively). The V-lines also reached higher total parasite densities than the ancestral parasites (C–D; *F*_1,22_ = 12.3, *p* = 0.002), but the C-lines and V-lines did not differ from each other (C–D; *F*_1,27_ = 1.6, *p* = 0.2).

Parasites passaged through AMA-1 vaccinated mice (V-lines) became more virulent than parasites passaged through sham-vaccinated mice (C-lines) (Figures 2 and 3). This difference had already arisen by the 10^th^ passage and was still apparent after 21 passages. Thus, in naïve mice, V-line parasites from both the 10^th^ and 21^st^ passage “generations” caused more anemia than their comparator C-lines (Figure 2A–B; Figure 3A–B; *F*_1,28_ = 8.4, *p* = 0.007, and *F*_1,27_ = 6.2, *p* = 0.02, respectively). The V-lines also induced more anemia than the parasites from which they were derived (passage 21 versus passage 0: *F*_1,22_ = 8.2, *p* = 0.008). After 20 passages, no changes in *ama-1* nucleotide sequence were detected in any of the lines (Figure S1). Thus, over the course of the experiment, parasites evolved in AMA-1 immunized mice became more virulent to naïve animals, and there was no evidence of nucleotide evolution at the *ama-1* target sequence.

The virulence differences apparent at the 10^th^ round of selection were associated with differences in parasite densities (Figure 2C–D). V-line parasites produced more parasites in total (Figure 2D; *F*_1,28_ = 11.5, *p* = 0.002), and had higher densities on the day of serial passage (*F*_1,28_ = 4.3, *p* = 0.04) than did C-line parasites. This is consistent with the hypothesis that selection by AMA-1 vaccination results in faster growing parasites, and that was why vaccine-evolved lines were more virulent. However, vaccine-adapted parasites from 21 passages, while still more virulent, did not achieve higher densities than C-line parasites (Figure 3D; V-lines versus C-lines: *F*_1,27_ = 1.6, *p* = 0.2), even though they did achieve higher densities than ancestral parasites (Figure 3D; passage 21 versus passage 0: *F*_1,22_ = 12.3, *p* = 0.002).

We performed another evaluation experiment, this time to compare the virulence and performance of V- and C-lines from passage 21 in AMA-1 vaccinated and sham-vaccinated mice (“evaluation experiment 4”). This allowed us to ask whether V-lines and C-lines were better adapted to the immune environment in which they evolved. Note that the half of this experiment conducted in sham-vaccinated mice closely replicates our previous evaluation of the virulence of the lines in naïve mice (“evaluation experiment 3”).

**Figure 4:**
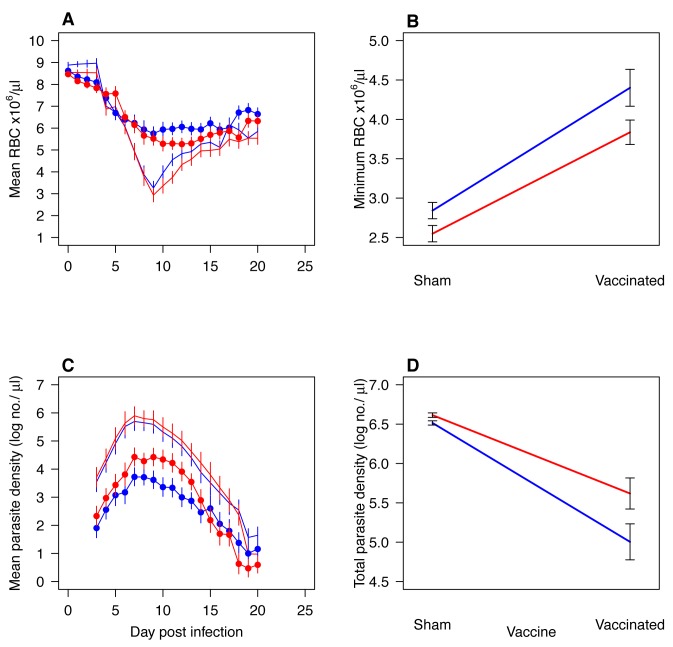
Virulence and densities of parasites that had been serially passaged 21 times in sham-vaccinated and AMA-1-vaccinated mice when assayed in sham-vaccinated or AMA-1-vaccinated mice (“evaluation experiment 4”). Curves (A and C) represents the kinetics (mean ±1 s.e.m.) of five C-lines (blue) and five V-lines (red) when assayed in sham-vaccinated (no symbol) or AMA-1-vaccinated (filled circles) mice. The interaction plots show the minimum RBC (B) and total asexual parasite densities (D) reached during infection of sham- or AMA-1-vaccinated mice with C-lines (blue line) or V-lines (red line). During infection of sham- and AMA-1-vaccinated mice, V-lines induced more anemia than C-lines (A–B; *F*_1,38_ = 4.0, *p* = 0.05 and *F*_1,38_ = 4.0, *p* = 0.05, respectively), but the magnitude was not significant (A–B; anemia, parasite×vaccination: *F*_1,76_ = 1.0, *p* = 0.3). V-lines and C-lines performed equally well in sham-vaccinated hosts (C–D: *F*_1,76_ = 1.0, *p* = 0.3), and although V-lines achieved higher densities in AMA-1-vaccinated hosts (C–D; *F*_1,38_ = 3.9, *p* = 0.05), the difference was not significant (Figure 4E–F; parasite×vaccination: *F*_1,38_ = 1.9, *p* = 0.1).

Again, we found that the V-lines were more virulent than the C-lines in control mice (Figure 4A–B; anemia *F*_1,38_ = 4.0, *p* = 0.05). This virulence difference was also apparent in vaccinated mice (Figure 4A–B; anemia *F*_1,38_ = 4.0, *p* = 0.05). The magnitude of the virulence difference was unaltered by the vaccine status of the host (Figure 4A–B; anemia, parasite×vaccination: *F*_1,76_ = 1.0, *p* = 0.3). Thus, vaccine-line parasites were more virulent in both sham- and AMA-1-vaccinated hosts.

If parasites had become adapted to the immune environment in which they evolved, we would expect V-lines to perform best in AMA-1-vaccinated hosts and C-lines to do better than V-lines in sham-vaccinated hosts. In fact, C- and V-lines did equally well in sham-vaccinated hosts (Figure 4C–D: *F*_1,38_ = 1.9, *p* = 0.1), just as they did in naïve mice in evaluation experiment 3 (Figure 3). The V-lines did achieve higher densities in AMA-1-vaccinated hosts (Figure 4C–D; *F*_1,38_ = 3.9, *p* = 0.05), as expected if indeed the V-lines were better adapted to vaccinated hosts, but this difference was itself not significantly different from that observed in sham-vaccinated hosts (Figure 4C–D; parasite×vaccination: *F*_1,76_ = 2.8, *p* = 0.09).

## Discussion

The main evolutionary concern of malaria vaccine developers is that antigenic escape will erode vaccine efficacy [7],[21]–[27]. Evolutionary biologists have raised a different concern, suggesting that some vaccines may drive the evolution of more virulent pathogen variants [28]–[37]. Virulence evolution would put unvaccinated individuals at risk of more severe disease should they become infected. In this study we used serial passage experiments in mice to test whether the candidate malaria blood-stage vaccine AMA-1 creates within-host conditions that selectively favor the emergence of more virulent parasite variants. In three separate phenotyping experiments, we found that parasites selected by passage through AMA-1-vaccinated mice caused more severe disease, removing 20% more RBCs in unvaccinated hosts than did the parasites evolved in unvaccinated mice (Figures 2–4, panels A and B). Importantly, vaccination did not select for antigenic escape at the *ama-1* locus (Figure S1). Our data highlight the importance of considering the evolutionary repercussions of blood-stage vaccines. These vaccines evidently have the capacity to cause changes at pathogen loci other than target antigens, including those responsible for disease severity.

In our experiments, all parasites were from the same clonal lineage, so variants differing in virulence must have been generated either by mutational processes or by switching of expression among members of multigene families. Presumably the more virulent variants had a relative fitness advantage during the process of serial passage and this was disproportionately larger in vaccinated hosts. Consistent with this, AMA-1-induced immunity controlled ancestral avirulent parasites more effectively than it controlled virulent descendant parasites (Figure 1). Virulent clones out-compete less virulent clones in mixed infections [73],[74]. This competitive advantage could be associated with more aggressive extraction of resources (e.g., RBCs) during infection or better performance in immune-mediated competition [49],[75]–[78]. We expect that comparative expression or genomic analyses of our different parasite lines will open up research programs that could shed light on the virulence determinants favored by AMA-1-induced immunity.

Our experiments highlight the importance of considering all types of evolution during malaria vaccine studies. To date, reports on parasite evolution in response to candidate vaccines in both human and animal trials have focused on antigenic polymorphism. But in the few cases where virulence correlates are also available, it is impossible to disentangle the effects of antigenic polymorphism from virulence. For instance, in a human field trial in Papua New Guinea with the *P. falciparum* “Combination B” blood-stage vaccine, which contained recombinant 3D7 MSP-2, the vaccine was less effective against parasites of the FC27 MSP-2 genotype. This was interpreted as reflecting a strain-specific protective response [56],[79] but could have also been because the FC27 MSP-2 genotypes were more virulent [80].

Results from an AMA-1 vaccine trial in non-human primates are also consistent with the possibility that more virulent *P. falciparum* strains are harder to control [81]. *Aotus* monkeys were vaccinated with AMA-1 derived from the *P. falciparum* 3D7 strain and then challenged with one of two heterologous strains, FVO or FCH/4. AMA-1 vaccination afforded less protection against the FVO strain [82]. This could have been because of greater epitope dis-similarity between the AMA-1 of FV0 and 3D7 strain [81] or because of the greater virulence of FVO parasites [82].

### Caveats

Our data show that immunization with a recombinant malaria vaccine can create ecological conditions that favor parasites that cause greater disease severity in unvaccinated individuals. But we are a long way from being able to assess the likelihood of this occurring in human malaria populations, were a malaria vaccine to go into widespread use. Most obviously, generalizing from animal models is notoriously difficult in malaria (reviewed in this context by [76],[83]), so extreme caution is warranted. But in addition to this generic issue, many potentially important considerations remain to be evaluated. Some of these are the following.

First, in human populations there will be variation in levels of immunity due to prior infection. Whether existing natural immunity will act to enhance or suppress vaccine-imposed selection for more virulent parasite variants remains to be determined. In mice, live parasite-induced immunity [30] and AMA-1-induced immunity (this study) both promote the evolution of virulence. Further experiments are needed to determine whether both occurring together in the same host would further promote virulence or whether the effects might be less than additive. It could be argued that semi-immune individuals will already naturally be imposing selection for greater virulence in the field, and the effects of vaccination will be no worse. However, the aim of vaccination programs is to increase the number of immune people in a population, and if that is achieved, a greater proportion of the parasite population will be evolving in immune hosts.

Second, our data show that virulence rises with serial passage, as it does in many systems [51]. In nature, something must counter within-host selection for virulence (or all pathogens would be extremely virulent). It has been hypothesized that syringe passage, which by-passes natural transmission, eliminates this counter-selection against excessive virulence that arises through host death [51]. This must be true in the limit, but the virulence increases we observed here as a consequence of immunity are likely to be far from this limit because mouse death played no role in the selection process in our serial passages (Figure S2). In the *P. chabaudi*-mouse model, more virulent infections are more infectious to mosquitoes [35],[36], and serial passage enhances virulence and transmission stage production [30],[52]. Virulence differences generated by experimental evolution using protocols identical to ours, but using whole-parasite immunized mice rather than a recombinant antigen, were not eliminated by mosquito transmission [30],[84]. If within- and between-host selection on virulence are somehow antagonistic, an important question is how they play out in the field now, and how vaccination might affect that. Our data show that the within-host selection for virulence is strengthened by vaccine-induced immunity.

Third, our experimental design involved passaging parasites every 7 d. We chose that timing because that is after a period of rapid parasite population expansion (selection) but before naïve mice begin mounting a strong acquired response against malaria [49],[85]–[90]. This meant that, in contrast to parasites in our vaccinated mice, our control-selected lines were under only modest antibody-mediated immunity. Without further experimentation, it is unclear whether onward transmission on any other days would lead to more or less potent selection on virulent variants. Later passage could select for parasite variants that are even more resilient against the mounting immune response; earlier passage may relax selection against competitively less able variants. How that would play out in terms of transmission to mosquitoes summed over the whole infectious period remains to be determined.

### Conclusions

Our data demonstrate that immunity induced by a recombinant antigen that is a candidate for human malaria vaccines can increase the potency of within-host selection for more virulent malaria parasites. In contrast, we found no evolution of the parasite locus controlling production of the target antigen. This does not exclude antigenic polymorphism as a challenge for vaccine efficacy, nor does it mean that virulence evolution is inevitable in populations immunized with a leaky (non-sterilizing) vaccine. But it does argue that a range of evolutionary trajectories are possible in response to vaccination [36],[44], and that epitope evolution is not the only evolution that can occur. We suggest that investigation of the impact on blood stage parasite densities and transmission should be a standard component of all Phase 3 malaria vaccine trials [10], and that whole genome analyses of parasites that survive and are transmitted from individuals in vaccinated and control arms in clinical trials should be a priority. Until there is a better understanding of the selection processes set up by imperfect vaccination, there is no reason to think that vaccine-driven evolution will occur only in genes encoding target antigens. Evaluating the medium term effects of widespread vaccination (evolutionary risk) is a substantial challenge, not least because evolutionary change is likely to occur long after clinical trials have concluded (Box 1). More generally, there is little reason to think the vaccine-driven virulence evolution we have seen will be limited to malaria parasites. Analysis of virulence evolution in range of infectious diseases for which leaky vaccines are in widespread use would be of substantial interest.

**Box 1. Evaluating Evolutionary Risk**

Our experimental data demonstrate that widespread use of a malaria vaccine *could* create parasites that cause more severe disease in unvaccinated individuals. However, it is not currently possible to evaluate the likelihood of such evolution. This is for a variety of reasons.

First, evolutionary trajectories in natural populations are always extremely difficult to predict from laboratory studies. Our experiments began with a single clone and relied entirely on mutational variation that arose during our experiments. The genetic variation in virulence and epitopes present in natural malaria populations are likely different. Malaria virulence is probably controlled by many genes, so that mutational variation in virulence may arise more frequently than escape variation in epitopes that are typically encoded by small genetic regions. That might be why we failed to see epitope evolution in our experiments but easily detected virulence evolution. If so, it is possible that attempts to broaden the response to AMA-1 with multivalent vaccines [26],[27],[98] might, by reducing the range of escape options available to the parasite, make virulence evolution more likely.

Even if we knew a lot about population-level genetic variation in virulence and epitopes, predicting evolutionary trajectories—and in particular evolutionary timescales—requires additional knowledge about genetic covariation with fitness. Virulence-transmission relationships, for example, are well understood in our mouse model [35],[36]. There is circumstantial evidence that similar relationships exist in *P. falciparum*, but the issue is far from settled and indeed may never be [35]. Additionally, we know very little about the strength of selection that will be imposed by candidate malaria vaccines. Clearly vaccine coverage will be an important determinant, but so too will the strength of vaccine-induced within host selection, which has yet to be estimated in people.

In our view, a profitable way forward is whole transcriptome comparisons of parasites that appear in people in vaccine and control arms of vaccine trials. And before novel vaccines go into widespread use, it should be a high priority to collect random samples of parasites from the pre-vaccine era and then to regularly collect random samples perhaps every 5 years after that. Whole transcriptome analyses of longitudinal parasite samples have the potential to detect vaccine-driven evolution of virulence determinants.

## Material and Methods

### Ethics Statement

This study was carried out in strict accordance with the recommendations in the Guide for the Care and Use of Laboratory Animals of the National Institutes of Health. The protocol was approved by the Animal Care and Use Committee of the Pennsylvania State University (Permit Number: 27452).

### Parasites and Hosts

We used the DK clone of *P. chabaudi adami*, which was originally collected from thicket rats (*Thamnomys rutilans*) in the Congo Brazzaville [91]–[93], and subsequently cloned by limiting dilution. Laboratory genotypes are stored as stable isolates in liquid nitrogen with subscript codes used to identify their position in clonal history [52]. Mice in our experiments were female C57Bl/6, at least 6–8 wk old. Parasite densities were estimated from day 4 from samples of tail blood using Giemsa-stained thin smears and red blood cell density was estimated from day 0 by flow cytometry (Beckman Coulter), or by genotype-specific real-time quantitative real-time PCR (qPCR) assays as described previously [74]. For amplification of the DK genotype, we used the forward primer previously used to amplify AS/AJ genotypes [74] and the DK genotpe-specific reverse primer 5′ GATTGTAGAGAAGTAGAAAATACA GATACAACTAA 3′.

### Vaccination

All mice were in one of the following three immune classes: naïve (never vaccinated with the adjuvant or the AMA-1 antigen), sham-vaccinated (which were immunized with adjuvant alone), or vaccinated (which were immunized with AMA-1 antigen plus adjuvant). We use that terminology consistently throughout.

Immunization protocols were similar to those described by Anders and others [53],[94],[95]. Briefly, vaccination was with the ectodomian of the AMA-1 protein derived from *P. c. adami* genotype DK [53]. AMA-1 was emulsified with Montanide ISA 720 adjuvant (Seppic). Each mouse was injected intra-peritoneally with a total of 10 µg of protein on two occasions with a 4-wk interval. Sham-vaccinated mice were injected with Montanide ISA720 plus PBS. During serial passage, and during the evaluation experiments, mice were infected with parasites 14 d after the second immunization.

### Serial Passages

We conducted two separate serial passage experiments (denoted A and B). All passages involved the syringe transfer of 0.1 ml of diluted blood containing 5×10^5^ parasites between mice every 7 d.

We first used serial passage simply to derive a more virulent parasite lineage from the ancestral DK (“serial passage experiment A”). This allowed us to test whether AMA-1-induced immunity controlled the derived (virulent) line less successfully than the ancestral (less virulent) line. *P. c. adami* genotype DK_294_ was derived via serial passage of ancestral *P. c. adami* genotype DK_122_ after a total of 30 passages though immunologically naïve mice.

The second serial passage (“B”) was the experimental evolution phase of our study (Figure S2). This was aimed at comparing the evolutionary consequences of passaging parasites through two contrasting selection treatments: sham- and AMA-1-vaccinated mice. We used sham-vaccinated mice so as to ensure that any evolved differences could be attributed to AMA-1 antigen, and not the adjuvant. We initially aimed to derive five independent parasite lines per selection treatment. At the start (generation 1), five mice that had been previously immunized with the AMA-1 vaccine (V- lines) or a sham vaccine (C-lines) were infected with *P. c. adami* genotype DK_247_ (generation 0) (Figure S2). Parasites from each one of the five mice at generation 1 were then used to infect at least two mice at generation 2 (forming a total of 10 sublines per treatment). Duplicate infections helped reduce the possibility of losing lines during the selection phase. Thus, from generation 2 to 21, parasites from each mouse within a selection treatment were used to infect a fresh mouse in the next generation. Some lines were lost (notably where AMA-1 vaccination induced a strongly protective anti-parasitic response) (Figure S2). When lines were lost, blood from a mouse in another line within that treatment group was used to infect at least two other mice in the next generation. This protocol ensured that at each generation 10 mice were infected with parasites within each selection treatment. A total of 410 mice were used during this experimental evolution phase.

### Virulence Phenotyping

Virulence and clone performance were assessed in four separate “evaluation” experiments conducted after the serial passages. In all cases frozen lines (*P. c. adami*-infected erythrocytes (IRBC)) were first introduced into naïve donor mice and then into naïve or sham-immunized experimental mice. Naïve donors are used because exact doses to initiate experiment infections cannot be obtained from frozen stock. Note that this single passage in naïve mice would, if it does anything, act to narrow the virulence differences observed in our experiments. Experimental mice were intra-peritoneally injected with 1×10^6^ IRBCs.

Evaluation experiment 1 compared the performance of parasites derived from serial passage A with their pre-passage progenitors in vaccinated and naïve hosts (Table S1). Two mice died (one control immunized and one AMA-1 immunized both infected with derived parasites). These were included in the calculation of daily densities until death as death always occurred after the peak of infection (days 17 and 15, respectively).

Three further evaluation experiments were used to compare the virulence and parasites dynamics of the C-lines and V-lines from serial passage B (Table S1): evaluation experiment 2, parasites from passage 10 in naïve mice; evaluation experiment 3, parasites from passage 21 in naïve mice; and evaluation experiment 4, parasites from passage 21 in sham- and AMA-1-vaccinated mice. In these three evaluation experiments, we compared five surviving C-lines with five surviving V-lines, with each line used to infect three mice. The lines used and their history are as shown in Figure S2. In evaluation experiment 3, nine naïve mice were also infected with the ancestral lineage (*P. chabaudi* genotype DK_247_). During evaluation experiment 2, one mouse infected with C-line parasites died on day five and was thus excluded from all analyses

### DNA Sequencing and Sequence Analysis

To test selected parasites for epitope evolution, *ama-1* nucleotide sequences of the ancestral and derived parasites from experiment one and the ancestral, C- and V-line parasites from experiments 3 and 4 (passage 21 parasites) were established using a series of overlapping oligonucleotide primers designed by reference to the published sequences of *P. c. adami* DK [94],[95]. Parasite DNA was extracted as previously described [74]. AMA-1 was amplified as two gene fragments: Outer Forward 5′ CTTGGGTAATTGTTCCGA 3′ and Inner Reverse 5′ GCACTTCTAACCCTTTGGT 3′; Inner Forward 5′ GGGTCCAAGATATTGTAG 3′ and Outer Reverse 5′ GGGTTTCGTCTTTTCTAC 3′. PCR was performed using Nova *Taq* (Novagen), with the thermocycle profile; 95°C for 12 min, then 95°C for 1 min, 57°C for 1 min, and 72°C for 1 min (×30 cycles) ending at 72°C for 10 min. Amplified DNA was visualized on a 1% agarose gel and positive amplifications were cleaned with QIAquick Gel extraction kit (Qiagen) and sequenced in both directions with the same primers that were used for amplification. Sequencing was performed by Penn State DNA sequencing core facility and sequences were aligned and analyzed using ClustalW.

### Statistical Analysis

All analyses were conducted in R 2.10.1 [96]. All parasite density data were log transformed to meet normality assumptions of the models. For the analysis of evaluation experiments 2–4, which determined the consequences of evolution through sham- and AMA-1-vaccinated hosts (serial passage B), differences among sub-line variances (C-lines and V-lines) were first analyzed using mixed effect linear models with sub-line as a random effect [97]. In all experiments there were no sub-line variances with selection treatments so we only report the between-selection effects. For completeness, we report the more conservative analysis, based only on line means, in Table S2.

## Supporting Information

Figure S1 Nucleotide sequence of *P. chabaudi ama-1.* Consensus *P. c. adami ama-1* nucleotide sequence between the derived virulent parasites from used in “evaluation” experiment 1, the V-lines and C-lines used in “evaluation” experiments 3 and 4 (21 serial passages), and the ancestral lineages from which all lines were derived and all compared to the published *P. c. adami* DK *ama-1* (genebank accession number U49745). There was 100% *ama-1* sequence identity among and between all of the derived lines and with their ancestral lineages and to the published genebank sequence (shaded in grey). The outer forward and inner reverse primers used for amplification and sequencing are highlighted in bold and the inner forward and outer reverse primers are shown in lowercase lettering. All traces were examined by eye for multiple peaks, and none were observed. If parasites with base-pair changes were present in sequenced samples, they must have been there at frequencies less than about 20%.
(TIF)

Figure S2 Experimental evolution (serial passage B) in sham- and AMA-1-vaccinated animals. Schematic genealogy illustrating passage history of the C-lines and V-lines from the ancestral lineage. Nodes represent mice. To start, five mice that had been previously immunized with the AMA-1 vaccine or a sham vaccine were infected with *P. c. adami* genotype DK_247_ (passage 1) to initiate the V-lines and C-lines, respectively. Parasites from each one of the five mice at passage 1 were then used to infect at least two mice at passage 2 (forming a total of 10 sublines per treatment). From passage 2 to 21 parasites from each mouse within a selection treatment were used to infect a fresh mouse in the next passage. Where parasite lines were lost (filled red circles) blood from a mouse in another line within that treatment group was used to infect at least two other mice in the next generation. Lines were lost when parasite densities were below transmissible frequencies on day 7 PI either because of vaccine-induced immunity (V-lines) or errors in dose delivered to mice (C-lines). Diamonds represent parasite lines used in the different evaluation experiments.
(TIFF)

Figure S3 Parasite densities of each mouse during serial passage B in sham- and AMA-1- vaccinated animals. Each data point represents the log parasite density of each mouse in the C-lines (blue circles) or V-lines (red triangles) from passage 1 to 21. Solid black lines represent the log linear regression change in parasite density per selection treatment over time.
(TIF)

Table S1 Description of evaluation experiments 1 to 4. V and C, V-or C-lines. Numbers indicate subline used. DK_122_, DK_247_, and DK_294_, DK ancestral genotypes with subscript codes used to identify their position in clonal history.
(DOC)

Table S2 Most conservative statistical analysis of evaluation experiments 2 to 4.
(DOC)

## Abbreviations

AMA-1: apical membrane antigen-1
RBCs: red blood cells
