# Supplementary figures and images for: The Evolutionary Consequences of Blood-Stage Vaccination on the Rodent Malaria Plasmodium chabaudi
Source: PLoS Biol. 2012 Jul 31;10(7):e1001368. doi: 10.1371/journal.pbio.1001368 (PMC3409122; doi:10.1371/journal.pbio.1001368)

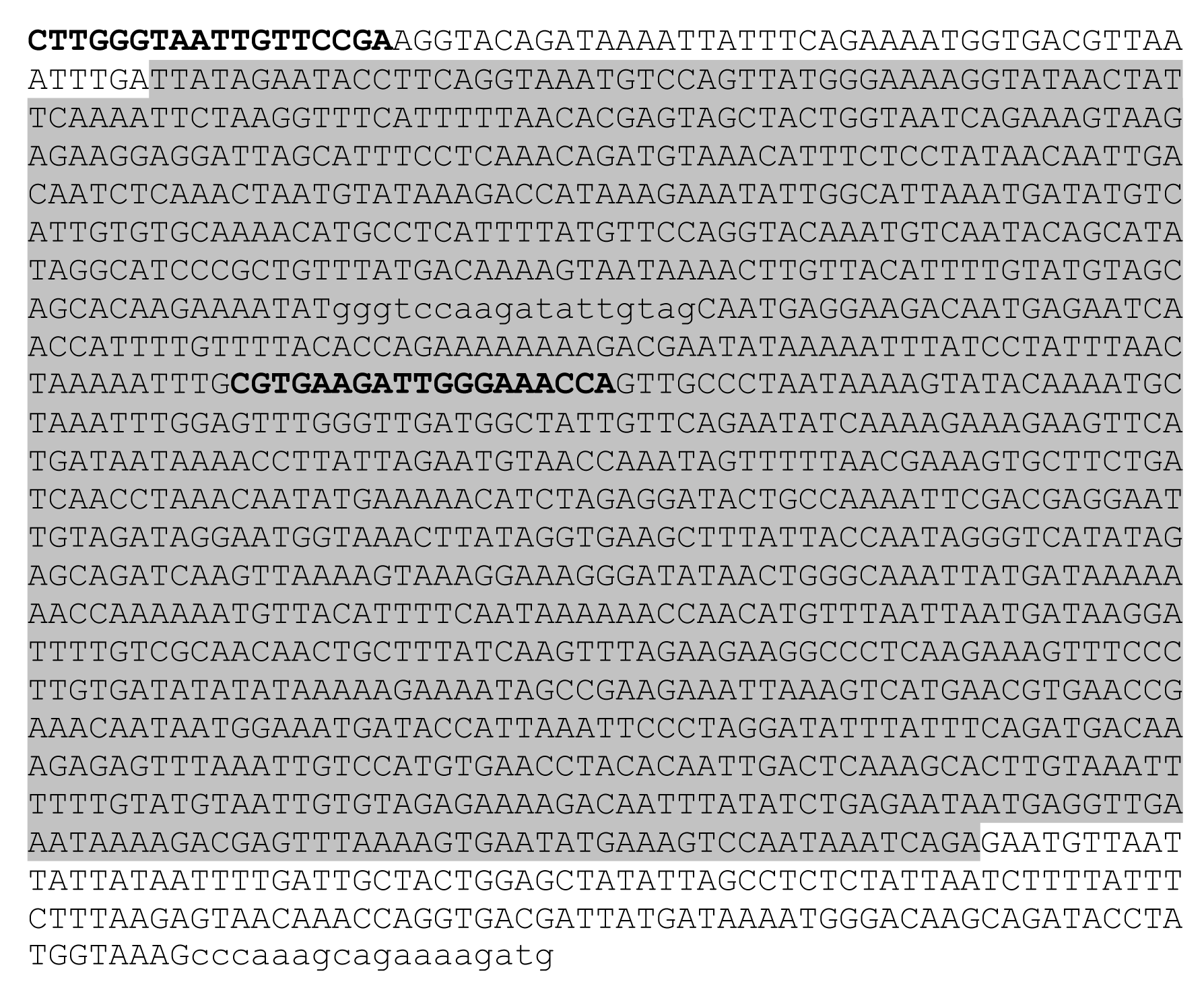

Supplement: Figure S1 — Nucleotide sequence of P. chabaudi ama-1. Consensus P. c. adami ama-1 nucleotide sequence between the derived virulent parasites from used in “evaluation” experiment 1, the V-lines and C-lines used in “evaluation” experiments 3 and 4 (21 serial passages), and the ancestral lineages from which all lines were derived and all compared to the published P. c. adami DK ama-1 (genebank accession number U49745). There was 100% ama-1 sequence identity among and between all of the derived lines and with their ancestral lineages and to the published genebank sequence (shaded in grey). The outer forward and inner reverse primers used for amplification and sequencing are highlighted in bold and the inner forward and outer reverse primers are shown in lowercase lettering. All traces were examined by eye for multiple peaks, and none were observed. If parasites with base-pair changes were present in sequenced samples, they must have been there at frequencies less than about 20%. (TIF) [file pbio.1001368.s001.tif]

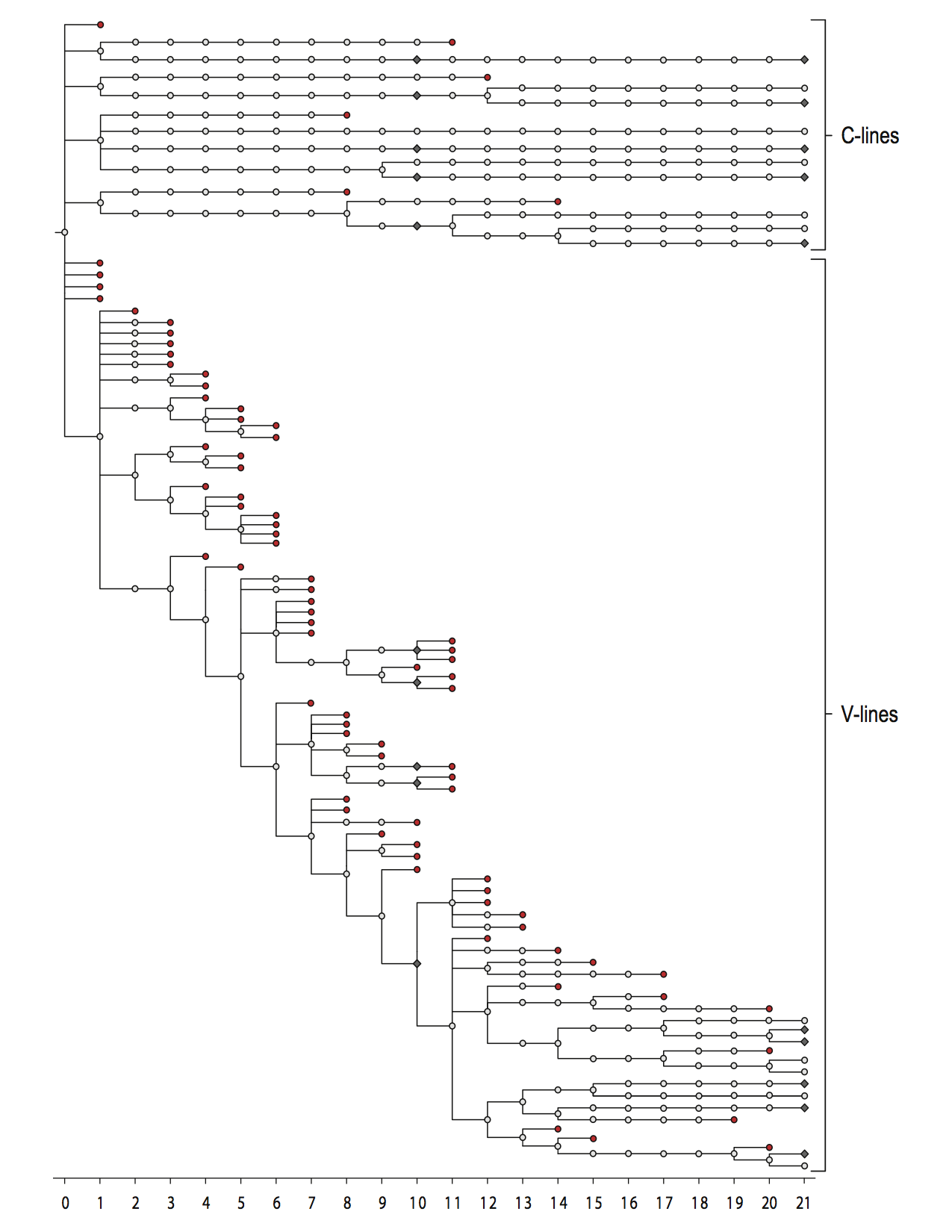

Supplement: Figure S2 — Experimental evolution (serial passage B) in sham- and AMA-1-vaccinated animals. Schematic genealogy illustrating passage history of the C-lines and V-lines from the ancestral lineage. Nodes represent mice. To start, five mice that had been previously immunized with the AMA-1 vaccine or a sham vaccine were infected with P. c. adami genotype DK247 (passage 1) to initiate the V-lines and C-lines, respectively. Parasites from each one of the five mice at passage 1 were then used to infect at least two mice at passage 2 (forming a total of 10 sublines per treatment). From passage 2 to 21 parasites from each mouse within a selection treatment were used to infect a fresh mouse in the next passage. Where parasite lines were lost (filled red circles) blood from a mouse in another line within that treatment group was used to infect at least two other mice in the next generation. Lines were lost when parasite densities were below transmissible frequencies on day 7 PI either because of vaccine-induced immunity (V-lines) or errors in dose delivered to mice (C-lines). Diamonds represent parasite lines used in the different evaluation experiments. (TIFF) [file pbio.1001368.s002.tiff]

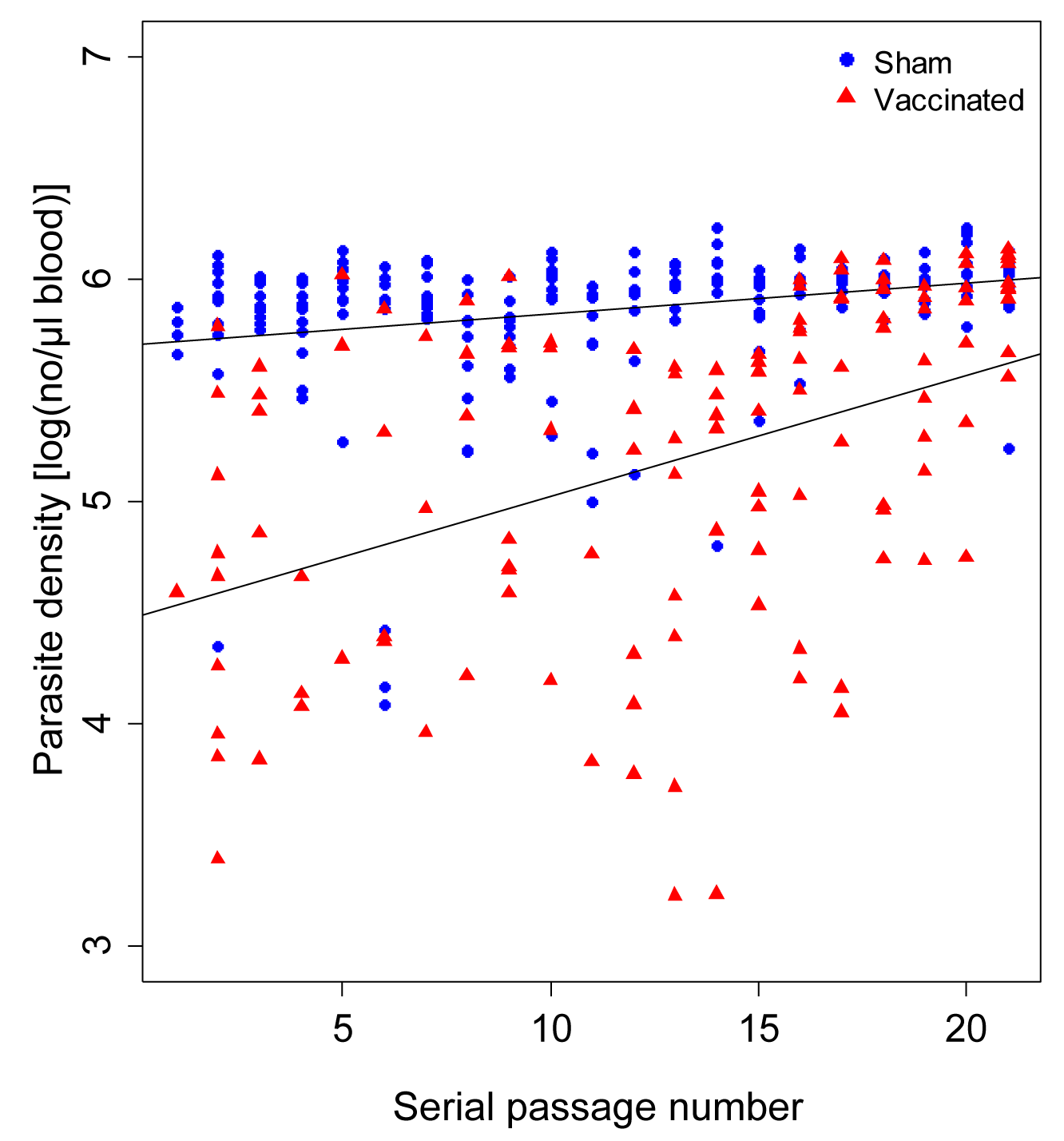

Supplement: Figure S3 — Parasite densities of each mouse during serial passage B in sham- and AMA-1- vaccinated animals. Each data point represents the log parasite density of each mouse in the C-lines (blue circles) or V-lines (red triangles) from passage 1 to 21. Solid black lines represent the log linear regression change in parasite density per selection treatment over time. (TIF) [file pbio.1001368.s003.tif]
