# Supplementary material for: The Evolutionary Consequences of Blood-Stage Vaccination on the Rodent Malaria Plasmodium chabaudi
Source: PLoS Biol. 2012 Jul 31;10(7):e1001368. doi: 10.1371/journal.pbio.1001368 (PMC3409122; doi:10.1371/journal.pbio.1001368)
Supplement: Table S1 — Description of evaluation experiments 1 to 4. V and C, V-or C-lines. Numbers indicate subline used. DK122, DK247, and DK294, DK ancestral genotypes with subscript codes used to identify their position in clonal history. (DOC) [file pbio.1001368.s004.doc]

| **Evaluation exp** | **Immune status** | **Parasites** | **Subline** | **No. mice** |
| --- | --- | --- | --- | --- |
| 1 | Sham-vaccine | Ancestral | Ancestral (DK_122_) | 4 |
|  | Sham-vaccine | Derived | Derived (DK_294_) | 4 |
|  | AMA-1 vaccine | Ancestral | Ancestral (DK_122_) | 4 |
|  | AMA-1 vaccine | Derived | Derived (DK_294_) | 4 |
| 2 | Naïve | Passage 10 | C2 | 3 |
|  | Naïve |  | C4 | 3 |
|  | Naïve |  | C6 | 3 |
|  | Naïve |  | C8 | 3 |
|  | Naïve |  | C10 | 3 |
|  | Naïve | Passage 10 | V1 | 3 |
|  | Naïve |  | V3 | 3 |
|  | Naïve |  | V4 | 3 |
|  | Naïve |  | V5 | 3 |
|  | Naïve |  | V10 | 3 |
| 3 | Naïve | Passage 21 | Ancestral (DK_247_) | 9 |
|  | Naïve |  | C1 | 3 |
|  | Naïve |  | C3 | 3 |
|  | Naïve |  | C5 | 3 |
|  | Naïve |  | C7 | 3 |
|  | Naïve |  | C10 | 3 |
|  | Naïve | Passage 21 | V2 | 3 |
|  | Naïve |  | V3 | 3 |
|  | Naïve |  | V6 | 3 |
|  | Naïve |  | V8 | 3 |
|  | Naïve |  | V9 | 3 |
| 4 | Sham-vaccine | Passage 21 | C1 | 3 |
|  | Sham-vaccine |  | C3 | 3 |
|  | Sham-vaccine |  | C5 | 3 |
|  | Sham-vaccine |  | C7 | 3 |
|  | Sham-vaccine |  | C10 | 3 |
|  | AMA-1 vaccine |  | C1 | 3 |
|  | AMA-1 vaccine |  | C3 | 3 |
|  | AMA-1 vaccine |  | C5 | 3 |
|  | AMA-1 vaccine |  | C7 | 3 |
|  | AMA-1 vaccine |  | C10 | 3 |
|  | Sham-vaccine |  | V2 | 3 |
|  | Sham-vaccine |  | V3 | 3 |
|  | Sham-vaccine |  | V6 | 3 |
|  | Sham-vaccine |  | V8 | 3 |
|  | Sham-vaccine |  | V9 | 3 |
|  | AMA-1 vaccine |  | V2 | 3 |
|  | AMA-1 vaccine |  | V3 | 3 |
|  | AMA-1 vaccine |  | V6 | 3 |
|  | AMA-1 vaccine |  | V8 | 3 |
|  | AMA-1 vaccine |  | V9 | 3 |
