# Supplementary material for: The Evolutionary Consequences of Blood-Stage Vaccination on the Rodent Malaria Plasmodium chabaudi
Source: PLoS Biol. 2012 Jul 31;10(7):e1001368. doi: 10.1371/journal.pbio.1001368 (PMC3409122; doi:10.1371/journal.pbio.1001368)
Supplement: Supplementary file 3 [file pbio.1001368.s005.doc]

Evaluation Exp.	Linear mixed effects test (line as random effect)	Result
2	Anaemia: V-lines versus C-lines in naïve mice	$F_{1,8}$= 8.3, p=0.02
	Total parasites: V-lines verusus C-lines in naïve mice	$F_{1,8}$=11.5, p=0.009
	Parasites on day 7: V-lines verusus C-lines in naïve mice	$F_{1,8}$= 3.6, p= 0.08
3	Anaemia: V-lines versus C-lines in naïve mice	$F_{1,10}$=6.2, p=0.03
	Anaemia: V-lines versus ancestral parasites in naïve mice	$F_{1,10}$=9.7, p=0.01
	Total parasites: V-lines verusus C-lines in naïve mice	$F_{1,10=}$ 1.1, p=0.3
	Total parasites: V-lines verusus ancestral parasites in naïve mice	$F_{1,10=}$ 7.3, p=0.02
4	Anaemia: V-lines versus C-lines in sham-vaccinated mice	$F_{1,8}$= 4.0, p=0.08
	Anaemia: V-lines versus C-lines in AMA-1 vaccinated mice	$F_{1,8}$= 2.2, p=0.1
	Anaemia interaction = Line*Vaccination	$F_{1,16}$= 0.5, p=0.4
	Total parasites: V-lines versus C-lines in sham-vaccinated mice	$F_{1,8=}$ 2.5, p=0.1
	Toatl parasites: V-lines versus C-lines in AMA-1 vaccinated mice	$F_{1,8=}$ 4.0, p=0.07
	Parasite interaction = Line*Vaccination	$F_{1,16=}$ 2.6, p=0.1
